# Correlation of Micro-Computed Tomography Assessment of Valvular Mineralisation with Histopathological and Immunohistochemical Features of Calcific Aortic Valve Disease

**DOI:** 10.3390/jcm9010029

**Published:** 2019-12-21

**Authors:** Guillermo Solache-Berrocal, Ana María Barral-Varela, Sheila Areces-Rodríguez, Alejandro Junco-Vicente, Aitana Vallina-Álvarez, María Daniela Corte-Torres, José Manuel Valdivielso, Juan Carlos Llosa, César Morís, María Martín, Isabel Rodríguez

**Affiliations:** 1 Cardiac Pathology Research Group, Instituto de Investigación Sanitaria del Principado de Asturias (ISPA), 33011 Oviedo, Spain; g.solache@ispasturias.es (G.S.-B.); cesarmoris@gmail.com (C.M.); 2 REDinREN from Instituto de Salud Carlos III (ISCIII), 28040 Madrid, Spain; valdivielso@irblleida.cat; 3 Cardiac Surgery Department, Complejo Asistencial Universitario de Salamanca (CAUSA), 37007 Salamanca, Spain; barralvarela@gmail.com; 4 Cardiology Department, Hospital Universitario Central de Asturias (HUCA), 33011 Oviedo, Spain; arecesrodriguez@gmail.com (S.A.-R.); ajuncovicente@gmail.com (A.J.-V.); juan_llosa@hotmail.com (J.C.L.); 5 Biobank of the Principality of Asturias, Hospital Universitario Central de Asturias (HUCA), Instituto de Investigación Sanitaria del Principado de Asturias (ISPA), 33011 Oviedo, Spain; alaicla@hotmail.es (A.V.-Á.); mdanielac@hotmail.com (M.D.C.-T.); 6 University Institute of Oncology of the Principality of Asturias (IUOPA), University of Oviedo, 33011 Oviedo, Spain; 7 Vascular and Renal Translational Research Group, Biomedical Research Institute of Lleida (IRBLleida), 25198 Lleida, Spain

**Keywords:** calcific aortic valve disease, aortic stenosis, valvular calcification, micro-computed tomography, histology, immunohistochemistry, macrophages, exosomes

## Abstract

Aortic valve stenosis is a serious disease with increasing prevalence in developed countries. Research aimed at uncovering the molecular mechanisms behind its main cause, aortic valve calcification, is thus crucial for the development of future therapies. It is frequently difficult to measure the extent of mineralisation in soft tissues and some methods require the destruction of the sample. Micro-computed tomography (µCT), a non-destructive technique, was used to quantify the density and volume of calcium deposits on cusps from 57 explanted aortic valves. Conventional and immunostaining techniques were used to characterise valve tissue degeneration and the inflammatory and osteogenic stage with several markers. Although most of the analysed cusps came from severe stenosis patients, the µCT parameter bone volume/tissue volume ratio distinguished several degrees of mineralisation that correlated with the degree of structural change in the tissue and the amount of macrophage infiltration as determined by CD68 immunohistochemistry. Interestingly, exosomal markers CD63 and Alix co-localised with macrophage infiltration surrounding calcium deposits, suggesting that those vesicles could be produced at least in part by these immune cells. In conclusion, we have shown that the ex vivo assessment of aortic valve mineralisation with µCT reflects the molecular and cellular changes in pathological valves during progression towards stenosis. Thus, our results give additional validity to quantitative μCT as a convenient laboratory tool for basic research on this type of cardiovascular calcification.

## 1. Introduction

Aortic valve stenosis is the most common cardiac valvulopathy in developed countries and its prevalence is expected to increase dramatically in the next decades as a consequence of population ageing [1]. For instance, a study has predicted that the number of individuals with severe aortic stenosis in Iceland may triple by the year 2060 [2]. Despite the challenge for national health systems, there still does not exist any pharmacological treatment that can halt or reverse the progression of the stenosis. Therefore, these patients must undergo a valve replacement procedure in order to restore normal blood flow. In fact, aortic valve replacement is the second most common cardiac procedure currently performed. Between 2014 and 2015, 18,000 of these operations were performed solely in Spain [3]. Research aimed at uncovering the pathological mechanisms behind aortic valve stenosis is, for these reasons, of vital necessity.

Calcification is the main contributor to aortic valve stenosis [4] and, in fact, stenosis constitutes the final stage of calcific aortic valve disease (CAVD), a tightly regulated process that resembles intimal artery calcification [5]. At the molecular level, damages to the valvular endothelium allow the infiltration of lipids which, upon oxidation [6,7], can promote an inflammatory process characterised by the formation of cellular infiltrates consisting mostly of macrophages, that is associated with remodelling of the tissue [8,9]. Cellular crosstalk involving extracellular vesicles such as exosomes is known to be crucial for the development of any type of cardiovascular calcification [10], and their production by the different cell types within the valve is thus concomitant to CAVD [11]. Analogous to the changes in vascular smooth muscle cells during artery calcification, interstitial cells of diseased valves experience osteogenic differentiation, with osseous metaplasia also being present in pathologic aortic valves [12,13]. The formation of mineral deposits is dominant during later stages, and the amount of calcium on the aortic valve has indeed been reported to correlate with the severity of aortic stenosis in patients [14,15,16]. Computed tomography technologies have been the gold standard for quantification of cardiovascular calcification for decades, although scientific publications using this sort of measurements to study CAVD in basic research remain scarce [17,18]. To the best of our knowledge, no studies have correlated the extent of calcification with changes at the protein level occurring in the valve tissue during calcification.

In spite of all that is known about CAVD, its pathobiology is still not fully understood. Also, the equivalence between valve and artery calcification is not total and the comparison of results from investigations in both fields is not always possible. Further research in the field is therefore needed to uncover the mechanisms behind disease progression. For this reason, the aim of our study was to assess the association between calcification, as measured by micro-computed tomography (μCT), and clinical, histological, and immunohistochemical parameters in patients with aortic valve dysfunction.

## 2. Experimental Section

### 2.1. Patients and Tissue Specimens

Patients subjected to aortic valve replacement surgery in the Hospital Universitario Central de Asturias (HUCA, Oviedo, Spain) between January and April 2014 were prospectively and consecutively recruited. Previous or suspected diagnosis of Marfan syndrome, Ehlers–Danlos syndrome or other connective tissue disorders associated with aortic valve disease, as well as rheumatic disease, were exclusion criteria. 

Established guidelines and criteria for the diagnosis of aortic stenosis, regurgitation and ascending aortic aneurysm by echocardiographic analysis were used [19]. Anthropometric, demographic, and biochemical data were obtained pre-surgery according to routine laboratory practice. Hypertension was defined as the fulfillment of any of the following criteria [20]: clinical history of hypertension; systolic blood pressure ≥140 mmHg or diastolic blood pressure ≥90 mmHg, in at least two determinations; or antihypertensive treatment that was not administered as therapy to pathology other than arterial hypertension. Hyperlipidemia was defined according to the fulfillment of one of the following criteria [21]: clinical history of hyperlipidemia, total cholesterol levels ˃200 mg/dL, LDL cholesterol ≥130 mg/dL, HDL cholesterol ˂40 mg/dL or lipid lowering therapy. The existence of diabetes mellitus was considered based on the presence of any of the following premises [22]: history of diabetes mellitus accredited in a medical report, fasting blood glucose ≥200 mg/dL in any situation, and symptoms of diabetes mellitus, at least two fasting blood glucose determinations ≥126 mg/dL or current use of oral hypoglycemic treatments and/or insulin. Chronic kidney disease was defined following KDIGO Guidelines [23]. Tissue samples were preserved in RNAlater (Invitrogen, Waltham, MA, USA) in the operating room at the time of surgical valve replacement and then, one cusp from each valve was stored in 70% ethanol.

The study was conducted in accordance with the Declaration of Helsinki and the human sample collection protocol was approved by the Ethics Committee for Clinical Investigation of the Principality of Asturias (84/2013, project PI13/00497). All patients signed an informed consent form before enrolment. 

### 2.2. Micro-Computed Tomography

Calcium content of one aortic valve cusp from each patient was measured by μCT. Ethanol-preserved samples were analysed in a SkyScan 1174 high-resolution tomograph (Bruker, Billerica, MA, USA). All specimens were scanned at 50 kV of source voltage and 800 μA X-ray tube current. Around 1300 projection images from each sample were obtained with a rotation step of 0.3° and a frame rate of 2 for a 180° scan. An exposure time of 6200 ms was used and each scan lasted between 10 and 20 min depending on the size of the cusp. Flat-field correction was done at the beginning of each scan. 

Images were 3D reconstructed with NRecon software (Bruker, Billerica, MA, USA). The attenuation coefficient, beam hardening correction, smoothing and ring artefact reduction values were the same for all samples. The reconstructed images had a voxel size of 17.22 μm^3^. Morphometric analysis was next carried on CTAN software (Bruker, Billerica, MA, USA). The volume of interest was manually delimited on each sample and the threshold used for all of them was 0.74–3.39 g/cm^3^ of bone mineral density (BMD). This measurement was calibrated with two phantoms containing 0.25 and 0.75 g/cm^3^ of hydroxyapatite each.

Bone histomorphometry parameters BMD and bone volume/tissue volume ratio (BV/TV) were used as indicators of the amount of calcium deposited on the cusps [24]. Whereas BMD expresses the mean density at which hydroxyapatite is presented on a cusp’s total volume, BV/TV indicates what proportion of this volume is occupied by the mineral. 

### 2.3. Histological Analysis

Once μCT was performed on all samples, they were fixed in formalin, decalcified in a 10% formic acid solution and embedded in paraffin. To accommodate the cusps in the embedding cassettes, they were divided longitudinally into 3 or 4 pieces depending on their size. Thus, different regions of one sample were exposed on each paraffin block, allowing the visualisation of the valve tissue near the hinge, belly and free edge of the cusps. 

Routinely deparaffinised and hydrated 5 μm-thick tissue sections were stained following a standard haematoxylin and eosin protocol for the examination of their general morphology and the discrimination of the amorphous matrix, where calcium deposits were, from the rest of the tissue. In order to properly identify the different components of the extracellular matrix and the location of calcium deposits within them, an orcein staining was also performed following a protocol based on Unna-Taenzer’s method [25]. Briefly, an acidified 1% orcein solution was applied to the samples for 30 min. After rinsing with distilled water, the slides were stained with 0.3% indigo carmine in a saturated aqueous solution of picric acid. The excess of orcein was differentiated with a prolonged dehydration in absolute ethanol. In both cases, after the sections were dehydrated, coverslips were mounted with Entellan (Merck, Darmstadt, Germany) for examination under light microscopy. 

Orcein staining served as the basis for the assignment of cusps to a grade in Warren and Yong’s score according to their degree of structural change [26]. Cusps that kept the characteristic three-layer structure of their extracellular matrix unchanged or presented mild fibrosis were assigned to grade 1 of this score. Cusps in grade 2 presented, in contrast, small calcium deposits in the fibrosa layer, although their dimensions did not distort the architecture of the tissue. Cusps with calcium deposits whose size caused a prominent thickening of the fibrosa and spongiosa layers were assigned to grade 3, while deposits in grade 4 cusps also affected the ventricularis layer, thus featuring discontinuities in elastic fibres.

### 2.4. Immunohistochemistry

Tissue sections were also deparaffinised and hydrated for immunohistochemical analysis. Components of the Envision FLEX High pH kit (Dako-Agilent, Santa Clara, CA, USA) were subsequently used, with intermediate rinses in Phosphate Buffered Saline (PBS). When necessary, antigen retrieval was performed on a PT Link equipment (Dako-Agilent), in which sections were heated at 96 °C for 20 min using the kit’s Target Retrieval Solution, whose formulation also allows deparaffinisation and hydration. Antibodies against Alix (sc-53540, 1:50, Santa Cruz Biotechnology, Dallas, TX, USA), BMP2 (ab6285, 1:100, Abcam, Cambridge, UK), CD31 (GA610, “ready-to-use”, Dako-Agilent), CD63 (sc-5275, 1:200, Santa Cruz Biotechnology) and CD68 (M0876, 1:500, Dako-Agilent) were used. 3,3′-Diaminobenzidine (DAB) was used as a substrate for horseradish peroxidase-conjugated secondary antibodies. Sections were next counterstained with Mayer’s haematoxylin, dehydrated and mounted with Entellan. Positive and negative controls were performed for the optimisation of the different antibodies (Supplementary Figure S1).

Full slide scans were obtained with a SCN 400 Slide Scanner (Leica, Wetzlar, Germany). Images from randomly chosen fields were next taken from each scan with SlidePath Gateway software (Leica). All of the cusp portions were considered. An average of 20 images from areas representative of the whole length of the cusps were taken. The resolving power of these images was equivalent to that of a 20X objective. Fiji software [27] was employed for quantification of DAB-stained areas. The Colour Deconvolution plugin [28] was used to discriminate DAB’s brown from haematoxylin’s blue. After a minimum threshold for DAB intensity was set, the total stained area of each sample was estimated as the mean area of all the analysed fields. 

### 2.5. Statistical Analysis

All statistical analyses were carried out on SPSS Statistics software (Version 20, IBM, Armonk, NY, USA). Fisher’s exact test was used to compare frequencies of qualitative variables. Normality of quantitative variables was determined by a Kolmogorov-Smirnov test. Differences between means of normally distributed variables were analysed using either a two-tailed Student’s t test or ANOVA, depending on the number of groups that had to be compared. The degree of correlation between two normal variables was calculated by linear regression with Pearson’s coefficient. Spearman’s coefficient was used for this purpose when any variable was not normally distributed. Significance of trends between an ordinal independent variable and a continuous dependent variable was assessed by a Jonckheere-Terpstra test. *p*-values < 0.05 were considered significant.

## 3. Results

### 3.1. Population

A total of 57 patients met the specified criteria and had their valves collected. Main baseline characteristics of these patients are summarised in Table 1. Although different diagnoses were included in order to obtain tissue samples with a wide range of calcification levels, stenosis remained the most common one (67%).

### 3.2. Distribution of the μCT Measurements

**Figure 1:**
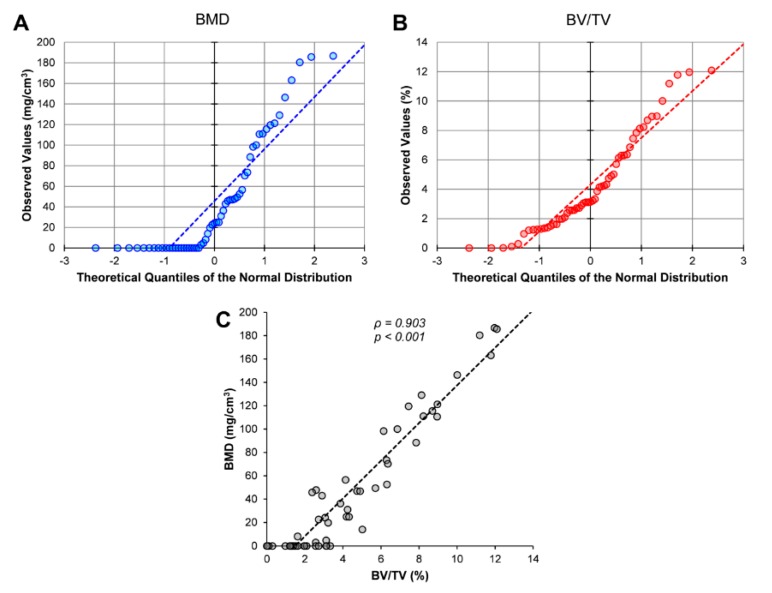
Distribution of BMD and BV/TV in the study population. Quantile-quantile plots of the bone mineral density (BMD) (**A**) and bone volume/tissue volume (BV/TV) (**B**) values obtained for the 57 cusps analysed by micro-computed tomography (μCT). (**C**) Correlation between BMD and BV/TV values. Statistical significance (*p*-value) of the correlation and Spearman’s coefficient (ρ) are shown.

μCT allowed the 3D reconstruction of aortic valve cusps (Supplementary Video S1) and the quantification of the extent of calcium deposits. The 57 aortic valve cusp samples effectively presented different levels of mineralisation, thus allowing a study of the complete process of valve calcification. Of the two variables used to express the extent of calcification, only BV/TV showed a normal distribution (Figure 1A, Kolmogorov-Smirnov test *p* = 0.014; Figure 1B, Kolmogorov- Smirnov test *p* = 0.108). There was, however, a strong positive correlation between BMD and BV/TV values (Figure 1C). Following this observation, we chose to use BV/TV as the only calcification variable in the subsequent analyses.

### 3.3. The Extent of Calcification as Measured by μCT is Different According to Clinical Characteristics of the Patients

Significant differences in the BV/TV values of cusps were found according to the diagnosis, valve anatomy and status of the ascending aorta of the patients (Table 2). On the other hand, no differences were found in the BV/TV value of cusps as a function of the patients’ sex or the presence of hypertension, hyperlipidaemia, diabetes or chronic kidney disease. There was no statistically significant correlation between BV/TV values and the patients’ age or echocardiographical parameters (all Pearson’s correlation *p*-values > 0.05).

### 3.4. The Extent of Calcification as Measured by μCT Correlates with the Degree of Structural Change according to Histology

**Figure 2:**
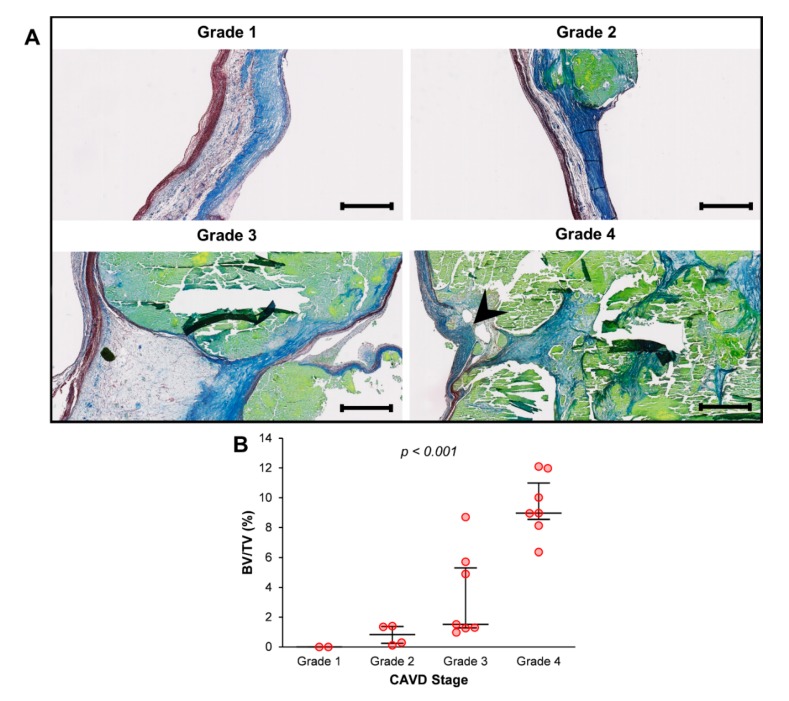
Relationship between micro-computed tomography measurements and the degree of valve tissue structural change: (**A**) Representative images of orcein-stained sections from each stage of calcific aortic valve disease (CAVD) according to Warren and Yong’s score. Collagen fibres were stained blue; elastin fibres, purple; and amorphous matrix from calcium deposits, light green. Arrowhead indicates a disruption in elastic fibres. Scalebar = 500 μm. (**B**) Bone volume/tissue volume (BV/TV) values for each stage of CAVD. *p*-value for trend (Jonckheere-Terpstra’s test) is shown. Median and interquartile range are represented.

A total of 20 aortic valve cusps situated at the extremes of the calcification spectrum according to bone histomorphometry parameters (the 10 most calcified and the 10 least calcified) were selected for subsequent histological examination. After light microscopy examination of stained slides, samples were assigned a grade in Warren and Yong’s score as an indicator of their degree of structural change. As shown in Figure 2A, and in accordance with previous observations [26], the cusps presented alterations that progressed in a sequential fashion from fibrosis to calcification and destruction of the tissue architecture. In fact, a Jonckheere-Terpstra test for ordered alternatives showed that there was a statistically significant trend of higher median BV/TV with higher Warren and Yong grades (from 1 to 4): T_JT_ = 133.000, *z* = 4.285, *p* < 0.001 for BV/TV (Figure 2B). Moreover, a strong positive correlation was found between BV/TV and Warren and Yong’s score (Spearman’s correlation: ρ = 0.879, *p* < 0.001).

### 3.5. Macrophage Infiltration Correlates with the Extent of Calcification as Measured by μCT

**Figure 3:**
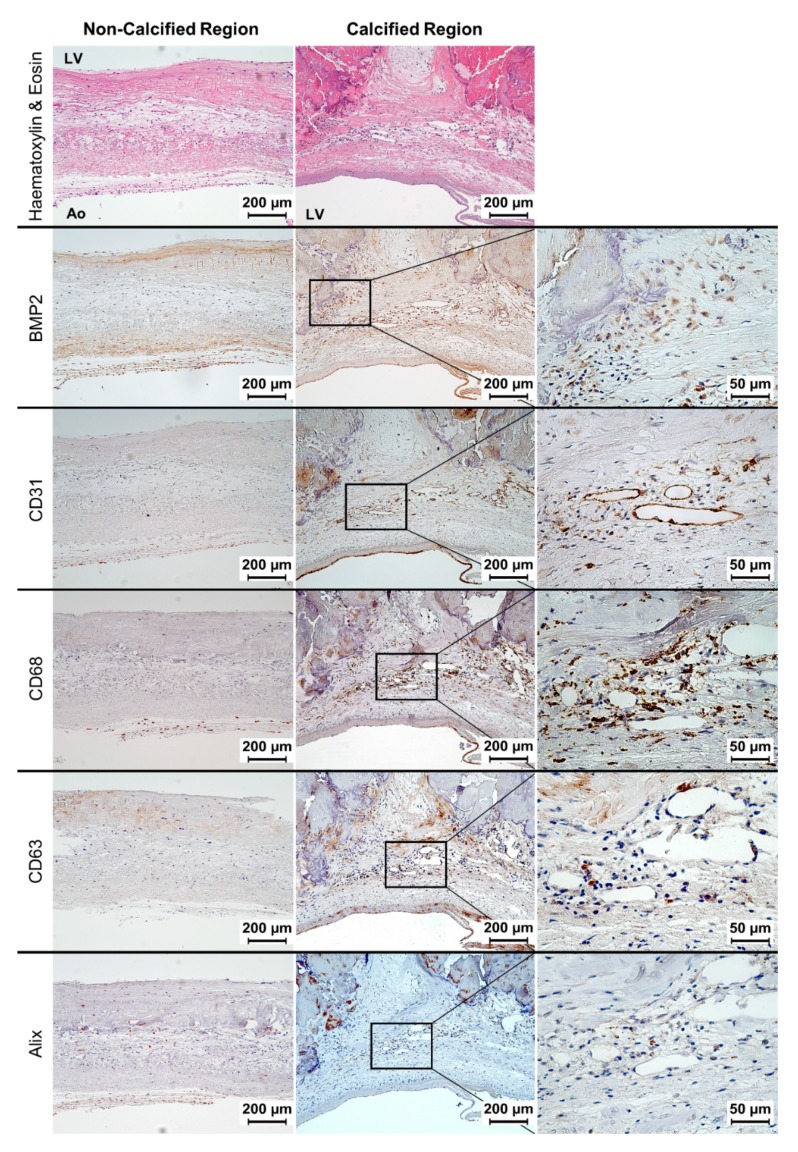
Representative images of immunohistochemistries for BMP2, CD31, CD68, CD63 and Alix on both calcified and non-calcified regions of aortic valve cusps. Images of a haematoxylin and eosin stain are shown to facilitate recognition of calcium deposits as acellular and eosinophilic areas with an amorphous extracellular matrix. LV = left ventricular side; Ao = aortic side.

Representative proteins of cellular processes that take place during CAVD were detected by immunohistochemistry on the 20 selected samples (Figure 3). Positivity for the osteogenic marker BMP2 was found in cells adjacent to calcium deposits. Classical endothelial cell marker CD31 was found near these deposits, indicating the presence of newly formed vessels. Abundant CD68-positive immunostaining could also be seen in calcified regions, thus identifying numerous cells around vessels as macrophages. CD63—a tetraspanin present on the membranes of exosomes [29]—and Alix—a protein involved in the biogenesis of these vesicles [30]—could be detected in the same areas. Non-calcified regions showed little or no positivity for these proteins.

**Figure 4:**
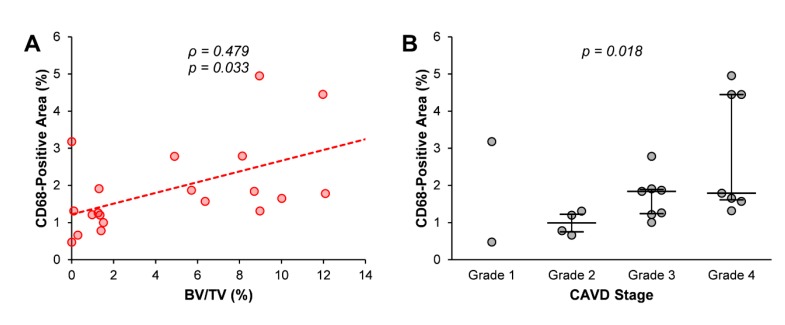
Relationship between macrophage infiltration and aortic valve calcification progression. (**A**) Correlation between CD68-positive area and bone volume/tissue volume (BV/TV) values. Spearman’s correlation coefficient (ρ) and statistical significance (*p*-value) are shown. (**B**) CD68-positive area in each stage of calcific aortic valve disease (CAVD) according to Warren and Yong’s score. *p*-value for trend (Jonckheere-Terpstra’s test) is shown. Median and interquartile range are represented.

The co-localisation of inflammatory infiltrates and exosomal markers in calcified regions could indicate that the production of extracellular vesicles by immune cells plays a central role in aortic valve calcification. In fact, a positive correlation between CD68-positive areas and BV/TV was found (Figure 4A). Simple linear regression showed a significant relationship between both variables (*p* = 0.017), with a slope coefficient of 0.145 and an R^2^ value of 0.278. Furthermore, a Jonckheere-Terpstra test for ordered alternatives showed that there was a statistically significant trend of higher median CD68-positive area with higher Warren and Yong grades (from 1 to 4): TJT = 105.000, *z* = 2.364, *p* = 0.018 (Figure 4B).

## 4. Discussion

Our results indicate that μCT assessment of valvular mineralisation permits not only the 3D localisation of calcium deposits, but also the correlation of the extent of calcification with histological and immunohistochemical findings in explanted aortic valves. Herein we show a progressive increase of BV/TV in aortic valve cusps with higher grades of tissue degeneration and a positive correlation of this parameter with the amount of macrophage infiltration. These data support the notion that μCT is of particular interest for the ex vivo study of aortic valve calcification.

While in vivo analysis of calcification is relevant for diagnostic purposes, ex vivo quantification is essential for mechanistic studies directed at uncovering the regulatory network behind disease progression. However, the latter is not often an easy task because of the destructive nature of some of the existing methods for the quantification of calcium in soft tissues [31]. μCT is a valid method for the study of calcification on explanted aortic valves that enables non-destructive quantification as well as 3D reconstruction of tissue structure. In fact, μCT is capable of calculating the density and volume of calcification within the tissue, an activity other radiographic methods such as dual-energy x-ray absorptiometry (DXA) cannot perform [32].

The examination of cardiac tissue by conventional histology and immunohistochemistry of previously μCT-scanned samples has already been performed with positive results, albeit on mice [33]. In our case, haematoxylin and eosin and orcein staining as well as immunostaining with anti-BMP2, CD31, CD68, CD63 and Alix antibodies yielded valuable information about CAVD progression at the tissue level. The increase of BV/TV with higher Warren and Yong grades gave physiological validity to the μCT measurements. In addition to this, the presence of BMP2-positive cells next to calcium deposits confirmed the implication of osteogenic differentiation and BMP signalling in valve calcification [34]. Immunohistochemistry for CD31 showed co-occurrence of neovascularisation and calcification, a fact that can be explained by the creation of a hypoxic environment in calcified valve cusps as a consequence of their structural remodelling and thickening, which in turn leads to increased angiogenesis. This hypothesis has been supported by the simultaneous overexpression of hypoxia-inducible factor 1-alpha and vascular endothelial growth factor in pathologic valve tissue [35]. Immunohistochemical positivity for CD31 also co-localised with that of CD68, a classical macrophage marker. A tight relationship between inflammation and neovascularisation has already been described in CAVD: newly formed vessels facilitate the infiltration of monocytes within the tissue and hence their differentiation into macrophages [8]. Co-localisation of CD68-positive areas with immunohistochemical positivity for both CD63 and Alix on calcified areas of aortic valve cusps could indicate that macrophages can direct CAVD progression through the production of exosomes with calcifying properties. The positive correlation between BV/TV and the percentage of CD68-positivity found in this work further supports this hypothesis. Although this mineralisation mechanism has been described for atherosclerotic lesions [36], we believe this is the first time that evidence for its contribution to CAVD has been gathered. Interstitial cells are to date the only valvular cell type that has been demonstrated to release exosomes in vitro under calcifying conditions [37] and, moreover, other valvular cell types apart from immune and interstitial cells could also be producing these vesicles. For instance, microparticles of endothelial origin have been found in arterial plaques [38]. The possible contribution of macrophage-derived exosomes to the development of aortic valve dysfunction needs further investigation. It might establish an additional link between CAVD and intimal artery calcification.

It is noteworthy that while the majority of the patients included in this study had severely stenosed aortic valves, the assessment of valvular mineralisation with μCT allowed for the distinction of several degrees of calcium deposition. Although this may represent a strength of the technique, it also implies that no correlation was evident between the extent of calcification and echocardiographical parameters. The fact that whole valves instead of single cusps were scanned in a study that reported such an association could be an explanation for this discrepancy [17]. On the one hand, μCT measurement of calcification on whole valves could provide useful information about the amount of mineral required to disturb normal functioning of these structures. On the other hand, the use of single cusps is a much simpler approach for the study of the mechanisms behind the progression of aortic valve calcification. Given that each cusp on one valve can have a different degree of calcification [39], the possibility that both calcium quantification and histological and immunoshistochemical analysis could be performed on the same cusp further illustrates the usefulness of µCT. Finally, and in spite of this study being performed on only one third of the aortic valve, we have found BV/TV to be correlated with some clinical features of the subjects included. This could be due to the fact that in patients with a severe form of stenosis, all cusps will be calcified despite the different rates at which this process progresses, so clinical translation of our findings is still possible.

Our experimental design has additional limitations that should be acknowledged. The primary disadvantage of the ex vivo study of calcified aortic valves is that patients undergo valve replacement only for severe and symptomatic forms of stenosis. The inclusion of samples from patients with mild and moderate forms of the disease would nevertheless provide interesting information about the initial phases of CAVD and their progression towards stenosis. Aortic valves from cadavers without signs of cardiovascular disease could serve as control tissues. Although this kind of specimen was not at our disposal, we believe the inclusion of patients subjected to valve replacement as a consequence of regurgitation—a disease not caused by calcification—could partially satisfy this need. The capacity of μCT to differentiate the extent of calcification even among severely diseased tissue samples partly overcomes this limitation. An example of this application could be the use of μCT quantification of calcium to assess the influence of gene polymorphisms on CAVD. We have, for instance, reported an association between the *MMP1* rs1799750 single nucleotide polymorphism and aortic valve calcification in surgical specimens using this method [40].

## 5. Conclusions

Our results support the validity of μCT measurement of valve mineralisation using bone histomorphometry parameters as a way of obtaining an objective calcium score that can be correlated with other variables, including molecular ones. Moreover, the numerical value provided my µCT is probably more accurate than those coming from in vivo measurements. µCT assessment is not affected by motion artifacts, does not require the use of contrast enhancement, and overlooks calcium deposited on adjacent regions. Thus, μCT yields valuable results that can be used in the pathophysiological study of CAVD. Given the increasing prevalence of this disorder, methods aimed at facilitating the scientific study of the calcification process are of paramount importance for the world population.

## Figures and Tables

**Table 1:** Baseline characteristics of the patients included in the study.

	Total	Type of Aortic Valve Disease			*p*-Value
		Regurgitation	Stenosis	Mixed	
	*N* = 57	*N* = 6	*N* = 38	*N* = 13	
Age, years	69.6 (11.1)	70.5 (7.6)	70.7 (10.8)	66.0 (13.1)	0.415
Sex					
Male	33 (57.9)	5 (83.3)	20 (52.6)	8 (61.5)	0.425
Female	24 (42.1)	1 (16.7)	18 (47.4)	5 (38.5)	
Valve Anatomy					
Tricuspid	41 (71.9)	6 (100.0)	27 (71.1)	8 (61.5)	0.188
Bicuspid	11 (19.3)	0 (0.0)	6 (15.8)	5 (38.5)	
Other	5 (8.8)	0 (0.0)	5 (13.2)	0 (0.0)	
BMI, kg/m^2^	29.6 (4.3)	29.3 (3.3)	29.8 (4.1)	29.3 (5.5)	0.913
Smoker Status					
Non-Smoker	35 (61.4)	2 (33.3)	24 (63.1)	9 (69.2)	0.108
Former Smoker	13 (22.8)	4 (66.7)	8 (21.1)	1 (7.7)	
Current Smoker	9 (15.8)	0 (0.0)	6 (15.8)	3 (23.1)	
Hypertension					
Yes	40 (70.2)	6 (100.0)	22 (57.9)	12 (92.3)	0.019
No	17 (29.8)	0 (0.0)	16 (42.1)	1 (7.7)	
Hyperlipidaemia					
Yes	25 (43.9)	3 (50.0)	17 (44.7)	5 (38.5)	0.923
No	32 (56.1)	3 (50.0)	21 (55.3)	8 (61.5)	
Diabetes					
Yes	11 (19.3)	1 (16.7)	8 (21.1)	2 (15.4)	1.000
No	46 (80.7)	5 (83.3)	30 (79.8)	11 (84.6)	
eGFR, mL/min/1.73 m^2^	76.3 (18.9)	63.9 (13.0)	76.1 (19.1)	83.0 (18.5)	0.121
Echocardiography					
Mean Gradient, mmHg	47.3 (15.0)	28.2 (25.1)	50.9 (11.6)	43.4 (15.6)	0.007
Peak Jet Velocity, m/s	4.3 (0.8)	3.2 (1.5)	4.5 (0.5)	4.2 (0.8)	0.002
LVEF					
Preserved	48 (84.2)	5 (83.3)	30 (79.8)	13 (100.0)	0.731
Mildly Reduced	2 (3.5)	0 (0.0)	2 (5.3)	0 (0.0)	
Moderately Reduced	5 (8.8)	1 (16.7)	4 (10.5)	0 (0.0)	
Severely Reduced	2 (3.5)	0 (0.0)	2 (5.3)	0 (0.0)	

Values from qualitative variables are presented as frequency (percentage) and comparisons between groups were performed using Fisher’s exact test. Values from quantitative variables are presented as mean (standard deviation) and comparisons between groups were performed using a two-tailed Student’s t test or ANOVA, as appropriate. BMI = Body mass index; eGFR = Estimated glomerular filtration rate; LVEF = left ventricle ejection fraction.

**Table 2:** Comparison of bone volume/tissue volume values according to several clinical characteristics of the patients.

	BV/TV	*p*-Value
	%	
Sex		
Male	4.0 (3.6)	0.379
Female	4.8 (2.9)	
Diagnosis		
Stenosis	5.1 (3.5)	0.006
Regurgitation	0.3 (0.5)	
Mixed	3.8 (2.3)	
Valve Anatomy		
Tricuspid	3.6 (3.0)	0.020
Bicuspid	6.7 (3.0)	
Other	4.9 (4.4)	
Ascending Aorta		
Normal	3.8 (3.0)	< 0.001
Dilated	8.7 (2.6)	
Hypertension		
Yes	3.6 (2.9)	0.070
No	6.0 (3.7)	
Hyperlipidaemia		
Yes	4.2 (3.5)	0.816
No	4.4 (3.2)	
Diabetes		
Yes	4.4 (3.3)	0.472
No	3.9 (3.6)	
Chronic Kidney Disease		
Yes	3.8 (3.5)	0.574
No	4.5 (3.3)	

Values are expressed as mean (standard deviation) and differences between groups were statistically analysed with a two-tailed Student’s t test or ANOVA, as appropriate. BV/TV = bone volume/tissue volume.

## Supplementary Materials

The following are available online at https://www.mdpi.com/2077-0383/9/1/29/s1. Figure S1: Positive and Negative Controls for Immunohistochemical Analysis; Video S1: 3D Reconstruction of a Calcified Aortic Valve Cusp Based on Micro-Computed Tomography.
