# Supplementary material for: Correlation of Micro-Computed Tomography Assessment of Valvular Mineralisation with Histopathological and Immunohistochemical Features of Calcific Aortic Valve Disease
Source: J Clin Med. 2019 Dec 21;9(1):29. doi: 10.3390/jcm9010029 (PMC7019701; doi:10.3390/jcm9010029)
Supplement: Supplementary file 1 [file jcm-09-00029-s001.zip › Supplementary_Figure_S1.pdf]

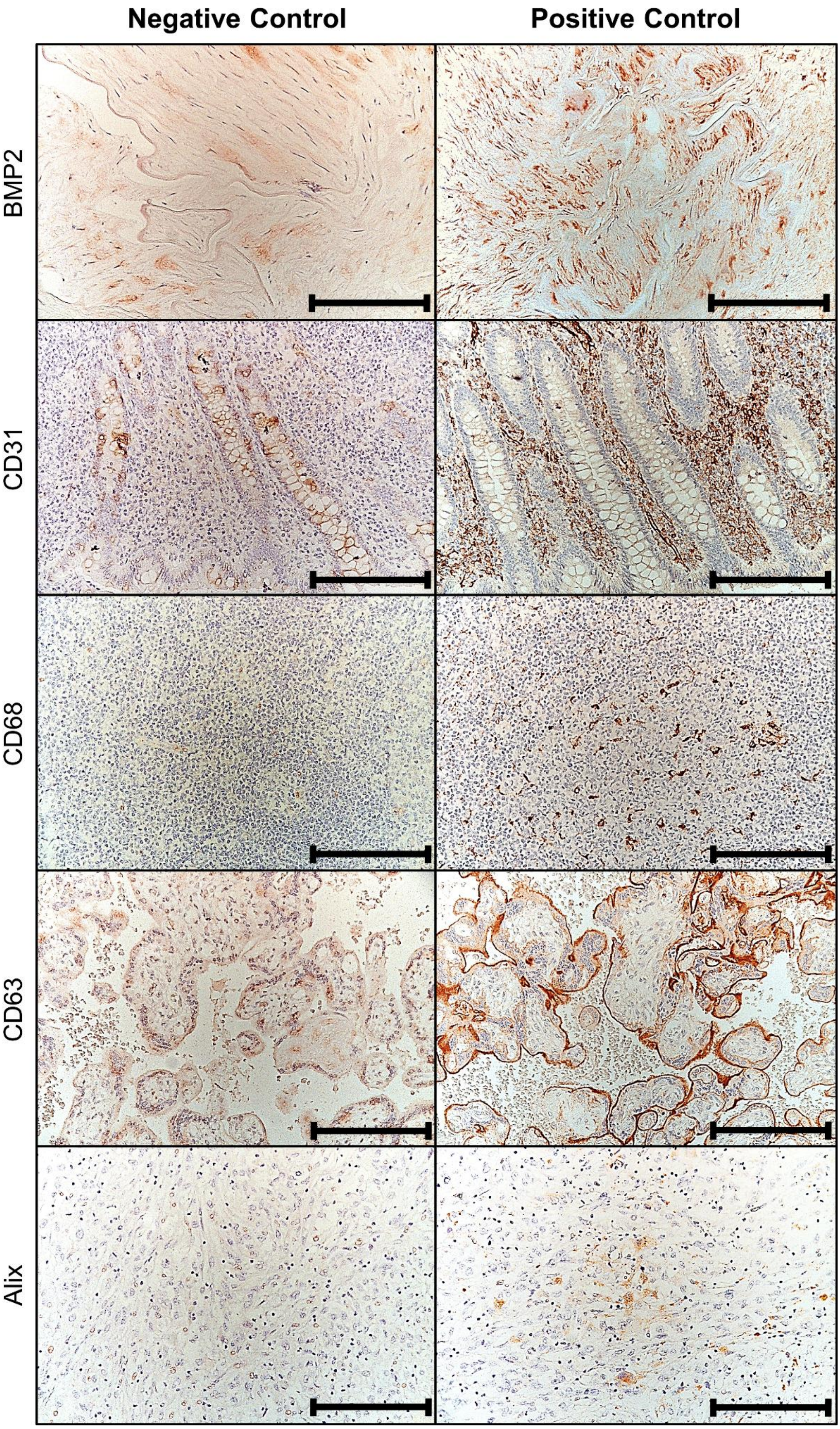

Supplementary Figure S1: Positive and negative controls for immunohistochemical analysis. Human tissues used for optimisation were pathological artery wall for BMP2, appendix for CD31, tonsil for CD68, placenta for CD63 and melanoma for Alix. Scalebar = 200 µm.
